# Pharmacological Characterization of [^3^H]CHIBA-3007 Binding to Glycine Transporter 1 in the Rat Brain

**DOI:** 10.1371/journal.pone.0021322

**Published:** 2011-06-23

**Authors:** Jichun Zhang, Jin Wu, Jun Toyohara, Yuko Fujita, Hongxian Chen, Kenji Hashimoto

**Affiliations:** Division of Clinical Neuroscience, Chiba University Center for Forensic Mental Health, Chiba, Japan; Rikagaku Kenkyūsho Brain Science Institute, Japan

## Abstract

Glycine transporter-1 (GlyT-1) in glial cells regulates extracellular levels of glycine, which acts as an obligatory co-agonist at the *N*-methyl-D-aspartate (NMDA) receptors in the brain. In the present study, we developed a novel radioligand, [^3^H]3-chloro-*N*-((*S*)-((*R*)-1-methylpiperidin-2-yl)(thiophen- 3-yl)methyl)-4- (trifluoromethyl)picolinamide ([^3^H]CHIBA-3007), for studying GlyT-1 in the brain. The presence of a single saturable high-affinity binding component for [^3^H]CHIBA-3007 binding to the rat brain membranes was detected. Scatchard analysis revealed an apparent equilibrium dissociation constant (K_d_) of 1.61±0.16 nM and a maximal number of binding sites (B_max_) of 692.8±22.8 fmol/mg protein (mean ± SEM, n = 3). The specific binding of [^3^H]CHIBA-3007 was inhibited by a number of GlyT-1 inhibitors, such as CHIBA-3007, desmethyl-CHIBA-3007, CHIBA-3008, SSR504734, NFPS/ALX5407, LY2365109 and Org24598, consistent with the pharmacological profiles of GlyT-1 inhibitors. Interestingly, the potency of eight GlyT-1 inhibitors (CHIBA-3007, desmethyl-CHIBA-3007, NFPS/ALX5407, LY2365109, Org24598, SSR504734, sarcosine, and glycine) for blocking *in vitro* specific binding of [^3^H]CHIBA-3007 was significantly correlated with the potency of these inhibitors for inhibiting [^14^C]glycine uptake in the rat brain membranes. In contrast, the GlyT-2 inhibitor ALX1393 exhibited very weak for [^3^H]CHIBA-3007 binding. Furthermore, the regional distribution of [^3^H]CHIBA-3007 binding in the rat brain was similar to the previously reported distribution of GlyT-1. The present findings suggest that [^3^H]CHIBA-3007 would be a useful new radioligand for studying GlyT-1 in the brain.

## Introduction

Glycine plays an important role in excitatory neurotransmission *via* strychnine-insensitive glycine sites located on the *N*-methyl-D-aspartate (NMDA) receptor [1]–[4]. In the central nervous system (CNS), synaptic levels of glycine are regulated by specific sodium/chloride-dependent transporters. The actions of glycine are terminated by reuptake via two high-affinity glycine transporters referred to as glycine transporter 1 (GlyT-1) and glycine transporter 2 (GlyT-2). GlyT-1 and GlyT-2 possess 12 putative transmembrane spanning domains, and share approximately 50% amino acid sequence identity [1]–[4]. GlyT-1 is widely expressed in the CNS, where it is present predominantly on glial cells. It is likely that GlyT-1 is responsible for glycine reuptake in forebrain areas, and in some regions it may be co-localized with strychnine-insensitive glycine sites on the NMDA receptor [5]–[11]. In contrast to GlyT-1, the distribution of GlyT-2 is predominantly neuronal and much more limited, being mainly restricted to the spinal cord, brainstem and cerebellum [9], [12]. Indeed, GlyT-2 is co-localized with strychnine-sensitive glycine receptors, suggesting that GlyT-2 may be a reliable marker for glycinergic neurons [1]–[4], [12], [13].

Accumulating evidence suggests that a dysfunction in glutamatergic neurotransmission via the NMDA receptors plays a role in the pathophysiology of schizophrenia [14]–[19]. Therefore, the potentiation of NMDA receptor function could provide a new approach for the treatment of neuropsychiatric diseases associated with NMDA receptor hypofunction. The effective therapeutic strategy is to increase synaptic levels of glycine by blocking the GlyT-1 in glial cells, because glycine is a co-agonist on the NMDA receptor [20]–[28].

Considering these results together, it would be of great interest to develop radioligands for studying GlyT-1 in the brain. Previously, two sarcosine-based GlyT-1 inhibitors, including [^3^H]NFPS [29], [^3^H](*R*)-NPTS [30], [31], have been reported. However, these two radioligands may be less suitable radioligands for studying GlyT-1 since these radioligands are non-competitive with respect to glycine [32], [33]. Recently, some non-sarcosine-based radioligands such as [^3^H]*N*-methyl-SSR504734 [31], [^3^H]SB-733993 and [^3^H]GSK931145 [33] have been developed.

**Figure 1:**
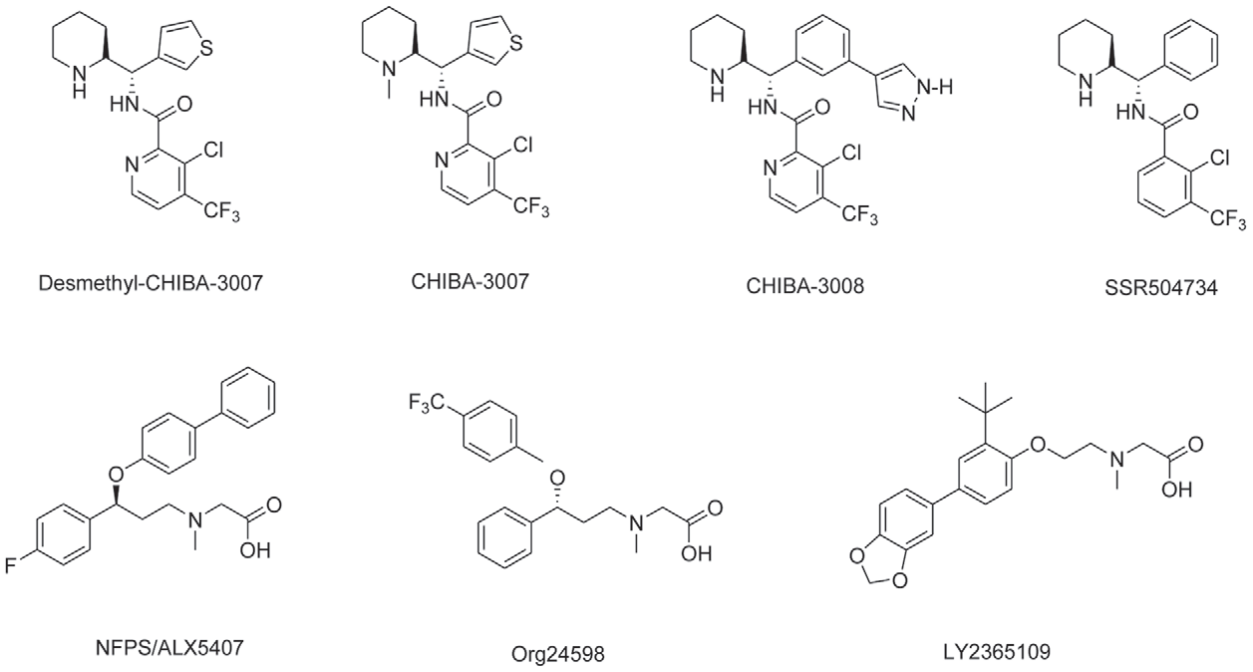
Chemical structure of GlyT-1 inhibitors used in this study.

3-Chloro-*N*-((*S*)-((*R*)-1-methylpiperidin-2-yl)(thiophen-3-yl)methyl)-4-(trifluoromethyl)picolinamide (CHIBA-3007)(Figure 1), is a novel non-sarcosine-based GlyT-1 inhibitor. We found that CHIBA-3007 was a more potent GlyT-1 inhibitor than SSR504734 (this study). In the present study, we report the characterization of a new radioligand [^3^H]CHIBA-3007 binding to GlyT-1 in the rat brain.

## Results

### Affinity and specificity of CHIBA-3007 for GlyT-1

The IC_50_ values of CHIBA-3007 and SSR504734 for [^14^C]glycine (10 µM) uptake in the rat brain membrane were 21.4±8.4 nM (n = 3, mean ± S.E.M.) and 84.5±29.8 nM (n = 3, mean ± S.E.M.), respectively. Thus, CHIBA-3007 was more potent than SSR504734 for glycine uptake inhibition. Furthermore, CHIBA-3007 (1 µM) was found to be devoid of activity (inhibition lower than 50%) for a 28 standard target binding profile **(** Table S1 **)**.

### Synthesis of [^3^H]CHIBA-3007

[^3^H]CHIBA-3007 was synthesized by methylation of the precursor (Figure S1). The radiochemical purity and specific activity of [^3^H]CHIBA-3007 were 98.9% and 2960 GBq/mmol (based on the specific activity of [^3^H]methyl iodide), respectively. The radiochemical yield of [^3^H]CHIBA-3007 was 13%.

### Equilibrium saturation binding of [^3^H]CHIBA-3007 to rat brain membranes

**Figure 2:**
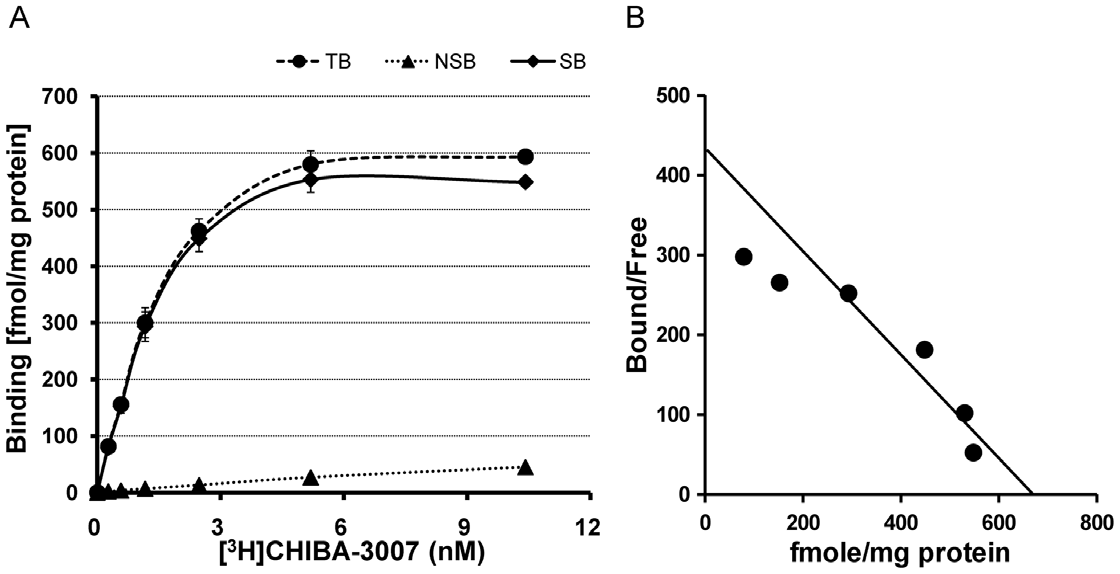
Specific binding of [^3^H]CHIBA-3007 to rat brain membranes. Rat brain membranes were incubated with various concentrations of [^3^H]CHIBA-3007 (0.3125–10 nM) for 120 min at room temperature. Nonspecific binding was estimated in the presence of 10 µM SSR504734. The results are from a typical experiment and values are the average of duplicate determinations. (A): The saturation binding isotherm shows specific binding of [^3^H]CHIBA-3007. TB: total binding, NSB: nonspecific binding, SB: specific binding. The results are means ± S.E.M. of three independent experiments performed in duplicate. (B): Scatchard plot analysis of [^3^H]CHIBA-3007 binding gave a K_d_ of 1.61 nM (95% confidence interval: 1.27 to 1.95 nM) and a B_max_ of 692.8 fmol/mg protein (95% confidence interval: 644.5 to 741.1 fmol/mg protein). The data are the mean of three independent experiments performed in duplicate.

For saturation-binding isotherms, 6 grade-diluted concentrations of [^3^H]CHIBA-3007 (0.3125–10 nM) were used. Specific binding of [^3^H]CHIBA-3007 to rat brain membranes was saturable and rapid, and represented >90% of total binding over the concentration range of [^3^H]CHIBA-3007 (Figure 2). In saturation-binding isotherms, nonlinear regression analysis of specific binding revealed an apparent K_d_ of 1.61±0.16 nM (95% confidence interval∶ 1.267 to 1.945 nM) and a B_max_ of 692.8±22.8 fmol/mg protein (95% confidence interval: 644.5 to 741.1 fmol/mg protein) (n = 3, mean ± S.E.M.) at room temperature (Figure 2).

### Pharmacological profiles of [^3^H]CHIBA-3007 binding to rat brain membranes

**Figure 3:**
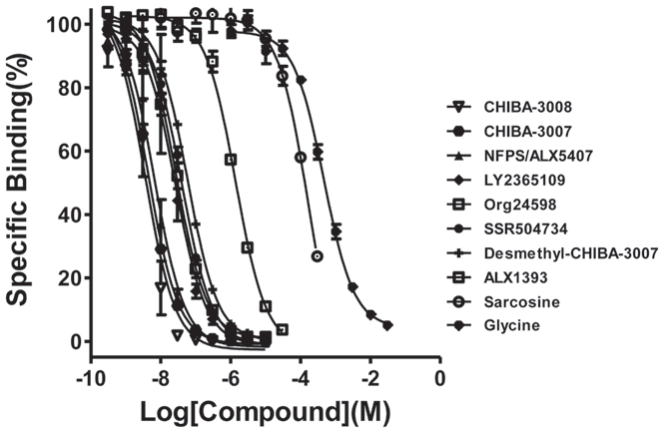
Competition curves of [^3^H]CHIBA-3007 binding by drugs. Inhibition curves for the displacement of [^3^H]CHIBA-3007 (1 nM) binding to rat brain membranes were obtained by 10 compounds such as CHIBA-3007, desmethyl-CHIBA-3007, CHIBA-3008, SSR504734, NFPS/ALX-5407, LY2365109, Org24598, glycine, sarcosine and ALX1393. The K_i_ denotes the affinity constant for binding to a single state of binding sites. The results are means ± S.E.M. of three independent experiments performed in duplicate.

The pharmacological inhibition of specific [^3^H]CHIBA-3007 (1 nM) binding to rat brain membranes was examined. Ten compounds, i.e., desmethyl-CHIBA-3007, CHIBA-3007, CHIBA-3008, SSR504734, NFPS/ALX5407, LY2365109, Org24598, glycine, sarcosine and ALX1393, were found to displace [^3^H]CHIBA-3007 binding to rat brain membranes (Figure 3). The Ki values of CHIBA-3008, CHIBA-3007, NFPS/ALX5407, LY2365109, Org24598, SSR504734 and desmethyl-CHIBA-3007 were 2.2, 2.8, 4.1, 16.2, 16.9, 24.6 and 35.9 nM, respectively (Table 1). The Ki values of the endogenous substances sarcosine and glycine for [^3^H]CHIBA-3007 binding were 103.5 µM and 287.9 µM, respectively. In contrast, the GlyT-2 inhibitor ALX1393 had very low affinity at [^3^H]CHIBA-3007 binding (851.7 nM)(Table 1).

**Table 1:** Drug inhibition of [3H]CHIBA-3007 binding to rat brain membranes.

Compounds	K_i_ (nM)
CHIBA-3008	2.2±0.6
CHIBA-3007	2.8±0.17
NFPS/ALX5407	4.1±0.9
LY2365109	16.2±4.9
Org24598	16.9±3.1
SSR504734	24.6±0.8
Desmethyl-CHIBA-3007	35.9±1.3
ALX1393	851.7±56.4
Sarcosine	103,492±10,572
Glycine	287,921±26,807

The inhibition of [^3^H]CHIBA-3007 binding by various drugs was determined with [^3^H]CHIBA-3007 (1 nM). Nine concentrations of the drugs were used for each determination. K_i_ values for the various drugs were determined as described in experimental procedures. The values represent the mean ± S.E.M of three independent experiments performed in duplicate.

**Figure 4:**
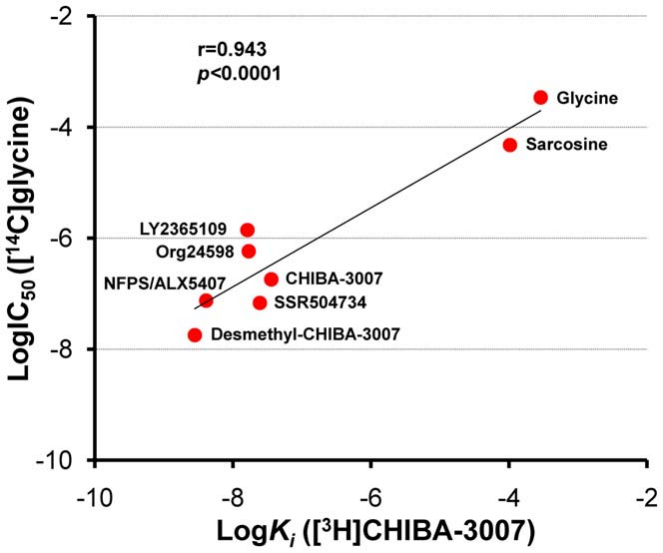
Comparison between the potency of various drugs for inhibiting [^3^H]CHIBA-3007 binding and [^14^C]glycine uptake into the rat brain membranes. Data are from Table 1 and Table 2. There was a significant (r = 0.943, *p*<0.0001) correlation between the potency for inhibiting of 8 compounds (desmethyl-CHIBA-3007, CHIBA-3007, NFPS/ALX5407, SSR504734, Org24598, LY2365109, sarcosine and glycine) for [^3^H]CHIBA-3007 binding and [^14^C]glycine uptake.

**Figure 5:**
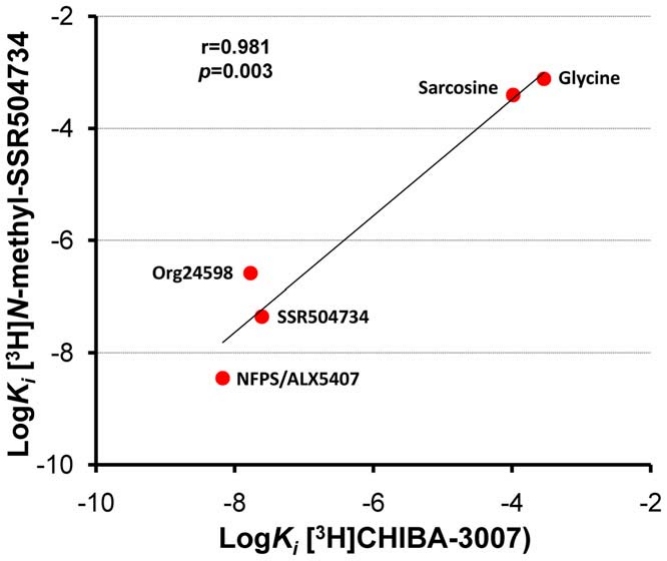
Comparison between K_i_ values of various drugs for inhibiting [^3^H]CHIBA-3007 binding and [^3^H]*N*-methyl-SSR504734 binding. Data of [^3^H]CHIBA-3007 are from Table 1. There was a significant (r = 0.981, *p* = 0.003) correlation between the potency for inhibiting of 5 compounds (NFPS/ALX5407, SSR504734, Org24598, sarcosine and glycine) for [^3^H]CHIBA-3007 binding and [^3^H]*N*-methyl-SSR504734 binding [31].

The potency of eight GlyT-1 inhibitors (CHIBA-3007, desmethyl-CHIBA-3007, NFPS/ALX5407, LY2365109, Org24598, SSR504734, sarcosine, and glycine) for blocking specific binding of [^3^H]CHIBA-3007 was significantly (r = 0.943, p<0.0001) correlated with that of these inhibitors for inhibiting [^14^C]glycine uptake in the same samples (Figure 4). Furthermore, there was also a significant (r = 0.981, p = 0.003) correlation between the potency for inhibition of [3H]CHIBA-3007 binding and the potency of drug inhibition for [3H]*N*-methyl-SSR504734 binding (data from [31]) (Figure 5).

**Table 2:** Drug inhibition of [^14^C]glycine uptake to rat brain membranes.

Compounds	IC_50_ (nM)
	[^14^C]Glycine uptake
CHIBA-3007	21.4±8.4
NFPS/ALX5407	83.7±30.3
SSR504734	84.5±29.8
Desmethyl-CHIBA-3007	187.2±40.0
Org24598	653.3±154.1
LY2365109	1,350±89.2
Glycine	338,766±18,285
Sarcosine	414,376±27,250

The inhibition of [^14^C]glycine uptake by various drugs was determined as described in the method. Nine concentrations of the drugs were used for each determination. The values represent the mean ± S.E.M of three independent experiments performed in duplicate.

### Regional distribution of [^3^H]CHIBA-3007 binding in the rat brain

**Figure 6:**
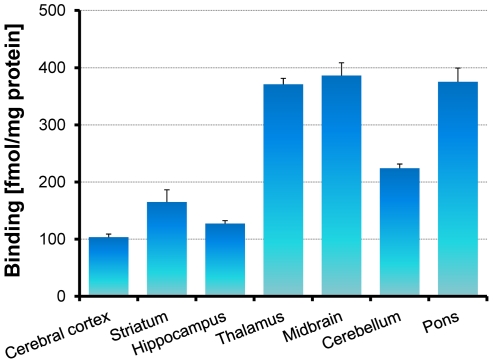
Regional distribution of [^3^H]CHIBA-3007 binding in rat brain. The regional distribution of [^3^H]CHIBA-3007 (1 nM) in the rat brain was determined. The radioactivity of the regions was in the order midbrain > pons > thalamus > cerebellum > striatum > hippocampus > cerebral cortex. The results are means ± S.E.M. of four independent experiments performed in duplicate.

The regional distribution of [^3^H]CHIBA-3007 binding in the rat brain is shown in Figure 6. Specific [^3^H]CHIBA-3007 binding was higher in the midbrain and lower in the cerebral cortex. The order of specific binding of [^3^H]CHIBA-3007 in the rat brain was as follows: midbrain > pons > thalamus > cerebellum > striatum > hippocampus > cerebral cortex. The regional distribution of [^3^H]CHIBA-3007 binding in the rat brain was similar to the distribution of GlyT-1 in the rat brain reported previously [6], [9], [10], [32].

## Discussion

The present study shows that [^3^H]CHIBA-3007, a non-sarcosine-based GlyT-1 inhibitor, is a novel and excellent radioligand for studying the pharmacology and distribution of GlyT-1 in the rat brain *in vitro*. The major findings of the present study are summarized as follows. First, the potency (IC_50_ = 21.4 nM) of CHIBA-3007 for inhibiting [^14^C]glycine uptake in the rat brain was higher than that (IC_50_ = 84.5 nM) of SSR504734, a potent, selective, and orally active GlyT-1 inhibitor [34]. Furthermore, CHIBA-3007 did not show any affinity (less than 50% at 1 µM) for a 28 standard target binding profile (Table S1), suggesting the high selectivity of CHIBA-3007 for GlyT-1. Second, the kinetic study showed that the specific binding of [^3^H]CHIBA-3007 to rat brain membranes reached equilibrium rapidly (less than 5 min, data not shown). Furthermore, Scatchard analysis showed that [^3^H]CHIBA-3007 selectively binds to rat brain with a high affinity (K_d_ = 1.61 nM). Nonspecific binding of [^3^H]CHIBA-3007 to rat brain membranes was very low (<10%).

Third, specific binding of [3H]CHIBA-3007 was inhibited by several compounds, including CHIBA-3008, CHIBA-3007, NFPS/ALX5407, LY2365109, Org24598, SSR504734, desmethyl-CHIBA-3007, sarcosine and glycine. Interestingly, there was a significant positive correlation between the Ki values obtained from the drug inhibition of [^3^H]CHIBA-3007 binding and the IC_50_ values obtained from drug inhibition of [^14^C]glycine uptake in the rat brain. Furthermore, we found a significant correlation between the potency of five compounds (NFPS/ALX5407, SSR504734, Org24598, sarcosine and glycine) for [^3^H]CHIBA-3007 binding and [^3^H]*N*-methyl-SSR504734 binding [31]. Finally, the regional distribution of [^3^H]CHIBA-3007 binding in the rat brain was consistent with previous reports on the localization of GlyT-1 mRNA and GlyT-1 protein using *in situ* hybridization, immunohistochemistry [6], [9], [10], and [35S](*S*)-2-amino-4-chloro-*N*-(1-(4-phenyl- 1-(propylsulfonyl)piperidin-4-yl)ethyl)benzamide: ACPPB) binding [32]. Taken all together, the present findings suggest that [^3^H]CHIBA-3007 binding sites could be associated with GlyT-1 in the rat brain.

Very recently, two non-sarcosine-based radioligands, [^3^H]SB-733993 and [^3^H]GSK931145, have been developed [33]. The potencies of SB-733993 (pIC_50_ = 7.20) and GSK931145 (pIC_50_ = 7.58) for inhibiting [^3^H]glycine uptake were more potent than that of SSR504734 (pIC_50_ = 6.52) [33]. [^3^H]SB-733993 and [^3^H]GSK931145 showed similar binding affinities for GlyT-1 and similar levels of specific binding. For both radioligands, the specific binding at concentrations around K_d_ values (1–2 nM) represented >90% of total binding [33], indicating low non-specific binding. However, the B_max_ values of both radioliagnds in the rat brain were higher (around 3000 fmol/mg protein) than the B_max_ values (692.8 fmol/mg protein) using [^3^H]CHIBA-3007. The reasons underlying this discrepancy are currently unclear. One possibility may be due to the differences in the methodology of sample preparation, and binding assay. Furthermore, non-sarcosine-based GlyT-1 inhibitors as well as glycine itself all showed competitive interactions with the binding of [^3^H]SB-733993 and [^3^H]GSK931145, whereas the sarcosine-based GlyT-1 inhibitors (NFPS/ALX5407 and Org25935) showed uncompetitive interactions with the bindings of both radioligands [33]. It is, therefore, likely that, similar to other non-sarcosine radioliagnds (e.g., [^3^H]SB-733993, [^3^H]GSK931145), [^3^H]CHIBA-3007 might bind to sites on the GlyT-1 that are orthosteric to the site at which glycine itself binds.

Previously, we reported that repeated administration of the NMDA receptor antagonist phencyclidine caused an increase of GlyT-1 protein as well as a reduction of extracellular glycine levels in the hippocampus, but not the frontal cortex [35]. The study suggests that increased GlyT-1 protein may play a role in removing the extracellular glycine in the synaptic cleft via GlyT-1, resulting in lower extracellular levels of glycine in the hippocampus [35]. To date, there has been no report about GlyT-1 density in the hippocampus of patients with schizophrenia, although it has been reported that GlyT-1 mRNA and protein levels were not altered in the prefrontal cortex and cerebellum of postmortem brain samples from patients with schizophrenia [36]. Therefore, it would be of interest to study whether levels of GlyT-1 are altered in the hippocampus of postmortem brain samples from schizophrenia using a [^3^H]CHIBA-3007-binding assay. Nonetheless, a further characterization of [^3^H]CHIBA-3007 binding in the postmortem brain sample from human is needed.

In conclusion, the present study shows that [^3^H]CHIBA-3007 binding sites are associated with GlyT-1 in the rat brain and that [^3^H]CHIBA-3007 could be a highly and specific and selective radioligand for studying GlyT-1 function in the brain *in vitro*.

## Materials and Methods

### Materials

CHIBA-3007, 3-chloro-*N*-((*S*)-((*R*)-piperidin-2-yl)(thiophen-3-yl)methyl)-4- (trifluoromethyl)picolinamide (desmethyl-CHIBA-3007), *N*-((*S*)-(3-(1*H*-pyrazol-4-yl)phenyl)((*R*)-piperidin-2-yl)methyl)-3-chloro-4-(trifluoromethyl)pyridine-2-carboxamide (CHIBA-3008: Taisho Pharmaceutical Ltd., Compound 86 [37], and SSR504734 (Figure 1) were synthesized by the previously reported methods [37], [38] with slight modification. CHIBA-3008 is a very potent inhibitor of GlyT-1 (IC_50_ = 0.2 nM for [^3^H]glycine uptake) [37]. The following drugs were obtained from the following sources: NFPS/ALX5407 (*N-* [3-(4-fluorophenyl)-3-(4-phenyl-phenoxy)propyl]-sarcosine) and LY2365109 ({[2-(4-benzo[1], [3]dioxol-5-yl-2- tert-butylphenoxy)ethyl]-methylamino}) sarcosine (Figure 1) were purchased from Tocris Bioscience (Bristol, UK); Org24598 ((*R*,*S*)-(±)*N*-methyl-*N*-[(4-trifluoromethyl)phenoxy]-3-phenylpropylglycine) (Figure 1), glycine, and *O*-[(2-benzyloxyphenyl-3-flurophenyl)methyl]-L-serine (ALX1393) were purchased from Sigma-Aldrich (St. Louis, MO). [^3^H]Methyl iodide (2.96 TBq/mmol) and [^14^C]glycine (3.96 GBq/mmol) were purchased from American Radiolabeled Chemicals Inc. (St. Louis, MO) and PerkinElmer Life & Analytical Sciences (Boston, MA), respectively.

### Synthesis of [^3^H]CHIBA-3007

[^3^H]CHIBA-3007 was synthesized by *N*-methylation of the desmethyl-CHIBA-3007 with [^3^H]methyl iodide (Figure S1). The 0.1 mL of [^3^H]methyl iodide toluene solution (370 MBq) was added to an ice-cold reaction vessel containing desmethyl-CHIBA-3007 (4 mg) and potassium carbonate (1.5 mg) in *N,N*-dimethylformamide (DMF, 0.3 mL). The reaction vessel was stirred at 0°C for 30 min. The reaction mixture was applied to a high performance liquid chromatography (HPLC) using an YMC Pack ODS-A column (10 mm in inner diameter ×250 mm in length; YMC Co., Ltd., Kyoto, Japan), comprised of UV absorbance (270 nm). A mixture of CH_3_CN/50 mM CH_3_COONH_4_/CH_3_COOH (350/650/3) was used as the mobile phase at a flow rate of 4 mL/min. The column eluent was collected automatically by using a fraction collector (Model 2110; Bio-Rad Laboratories, K.K., Tokyo, Japan) directly into polypropylene tubes. The 10-µL of each collected fractions were sampled into glass vials with 4 ml of scintillation cocktail (ACS-II; GE Healthcare Japan K.K., Tokyo, Japan). The radioactivity was determined using a liquid scintillation counter (LS-6500; Beckman Coulter, Tokyo, Japan). The radioactive fraction, eluted with a retention time corresponding to that of the authentic standard by was collected into an evaporation flask and evaporated to dryness. The residue was re-dissolved with 2 ml of ethanol. Chemical and radiochemical purity of [^3^H]CHIBA-3007 was analyzed by HPLC in a system consisting of a column (YMC-Pack Pro C18, 4.6 mm in inner diameter ×250 mm in length, YMC Co., Ltd., Kyoto, Japan), using CH_3_CN/50 mM CH_3_COONH_4_/CH_3_COOH (350/650/3) as a mobile phase at a flow rate of 1.0 ml/min.

### Preparation of Rat Brain Membrane

Male Crl: CD (SD) SPF/VF rats (8–10 week olds, 180–200 g)(Japan Charles River Inc., Tokyo, Japan) were used for the experiments. All animal studies were approved by the Animal Care and Use Committee of Chiba University (Permit Number: 22–122). All experiments were performed according to the Guidelines for Animal Experimentation and also conformed to the Guide for the Care and Use of Laboratory Animals published by the US National Institutes of Health. All efforts were made to minimize suffering.

After sacrificing the rats by decapitation, the brains were rapidly removed from the skulls. Whole brains or seven specific cerebral regions - the cerebral cortex, striatum, hippocampus, thalamus, midbrain, cerebellum and pons - dissected on ice by the method of Glowinski and Iversen [39] were stored at −80°C until use for the assay.

For the [^3^H]CHIBA-3007-binding assay, the tissues of whole brains or each specific brain region were homogenized in 15 volumes (w/v) of 10 mM 4-(2-hydroxyethyl)-1- piperazineethanesulfonic acid (HEPES) at pH 7.4 for 30 s on ice. The homogenate was centrifuged at 40,000 g for 15 min at 4°C. The supernatant was discarded and the pellet was re-suspended, homogenized and centrifuged as above. The membrane pellet was washed and re-suspended in ice-cold HEPES buffer and was then centrifuged three times. The final pellet was re-suspended in 15 volumes of the buffer (120 mM NaCl, 2 mM KCl, 1 mM CaCl_2_, 1 mM MgCl_2_ 10 mM HEPES, pH 7.5 at room temperature).

For [^14^C]glycine uptake, whole brains were homogenized in 10 volumes (w/v) of 0.32 M sucrose, buffered with 10 mM HEPES (pH 7.4). The homogenate was centrifuged at 1,000 g for 10 min to remove nuclei and debris, and then the supernatant was centrifuged again at 20,000 g for 20 min (synaptosomal P_2_ fraction). The pellet was washed and re-suspended in ice-cold 0.32 M sucrose, buffered with 10 mM HEPES (pH 7.4) and centrifuged again at 20,000 g for 20 min (washed P_2_ fraction). The pellet was re-suspended in 10 volumes of assay buffer with the following composition: 10 mM HEPES buffer (pH 7.4) containing 140 mM NaCl, 5.5 mM KCl, 1.8 mM CaCl_2_, 0.8 mM MgSO_4_, 5 mM glucose and 5 mM L-alanine (HB). The protein concentrations were measured by using a DC protein assay kit (Bio-Rad Laboratories Inc., Tokyo, Japan).

### [^3^H]CHIBA-3007 Binding Assay

Assays of the binding of [^3^H]CHIBA-3007 to rat brain membranes were performed. Aliquots of the rat brain membrane suspension (200 µL) were added in duplicate to a reaction mixture containing [^3^H]CHIBA-3007 and the indicated concentrations of test drug in a final volume of 0.5 mL. Non-specific binding was estimated in the presence of 10 µM SSR504734, a potent and selective GlyT-1 inhibitor [34]. [3H]CHIBA-3007 binding was allowed to occur for 120 min at room temperature for the equilibrium saturation and inhibition studies. The binding reaction was terminated by rapid vacuum filtration onto Whatman GF/B glass filters pretreated with 0.5% polyethyleneimine (Sigma-Aldrich Co.) using a 24-channel cell harvester (Brandell, Gaithersburg, MD). The filters were washed with 5 mL of ice-cold assay buffer 3 times, and placed in vials with 4 mL scintillation cocktail. The radioactivity trapped by the filters was determined using a liquid scintillation counter (Beckman LS-6500; Beckman Coulter K.K., Tokyo, Japan).

To examine the pharmacological profiles of [^3^H]CHIBA-3007 binding, ten compounds were used: desmethyl-CHIBA-3007, CHIBA-3007, CHIBA-3008, SSR504734, NFPS/ALX5407, LY2365109, Org24598, glycine, sarcosine and ALX1393.

### Inhibition of [^14^C]glycine Uptake

The assay of [^14^C]glycine uptake was started by adding 10 µM [^14^C]glycine to 200 µL of rat brain membrane in HB. The inhibition of [^14^C]glycine uptake by eight compounds—desmethyl-CHIBA-3007, CHIBA-3007, SSR504734, NFPS/ALX5407, LY2365109, Org24598, sarcosine and glycine—was performed for 15 min at 37°C as reported previously [34]. Non-specific uptake was estimated in the presence of 30 µM SSR504734. The uptake of [^14^C]glycine was terminated by rapid vacuum filtration onto Whatman GF/B glass filters pretreated with 0.5% polyethyleneimine. The filters were washed by buffer, and the radioactivity trapped by the filters was determined using a liquid scintillation counter as described above.

### Statistical Analysis

The data are shown as the mean ± standard error of the mean (S.E.M.). The dissociation constant (K_d_) and maximal binding (B_max_) values from saturation binding and the IC_50_ values from binding displacement by each drug were determined using the program GraphPad Prism (GraphPad Software, San Diego, CA). The K_i_ values were calculated from the IC_50_ values using the formula of Chung and Prusoff, K_i_ = IC_50_/(1+[L]/K_d_) [40], where the IC_50_ was the concentration that resulted in 50% inhibition of specific binding, [L] was the concentration of radioligand used and K_d_ was the dissociation constant. Correlation was analyzed by Pearson's Correlation Coefficient (PASW Statistics 19, Tokyo, Japan). Significance was set at *p*<0.05.

## Supporting Information

Table S1 Inhibition effect of CHIBA-3007 (1 µM) on radioligand binding to various receptors.
(DOCX)

Figure S1 Preparation of [^3^H]CHIBA-3007.
(TIFF)
