# Supplementary material for: Pharmacological Characterization of [3H]CHIBA-3007 Binding to Glycine Transporter 1 in the Rat Brain
Source: PLoS One. 2011 Jun 23;6(6):e21322. doi: 10.1371/journal.pone.0021322 (PMC3121759; doi:10.1371/journal.pone.0021322)
Supplement: Table S1 — Inhibition effect of CHIBA-3007 (1 µM) on radioligand binding to various receptors. (DOCX) [file pone.0021322.s001.docx]

Table S1. Inhibition effect of CHIBA-3007 (1 μM) on radioligand binding to various receptors

| Receptors | % Inhibition (1 μM) | | |
| --- | --- | --- | --- |
|  | CHIBA-3007 | Positive substance | Radioligand |
| Adenosine A1 | 2.12 | (DPCPX) | [^3^H]DPCPX |
| Adenosine A2 | 5.86 | (NECA) | [^3^H]CGS21680 |
| α1-Adrenergic (Non-selective) | 1.13 | (Prazosin) | [^3^H]Prazosin |
| α2-Adrenergic (Non-selective) | 20.98 | (Yohimbine) | [^3^H]RX821002 |
| β-Adrenergic (Non-selective) | 8.39 | ((±)-Propranolol) | [^3^H]DHA |
| Dopamine D1 | 2.32 | ((R)(+)-SCH23390) | [^3^H]SCH23390 |
| Dopamine D2 | 1.00 | ((+)-Butaclamol) | [^3^H]Spiperone |
| GABA -A (Agonist site) | 0.20 | (Muscimol) | [^3^H]Muscimol |
| GABA- A (BZ central) | 2.78 | (Diazepam) | [^3^H]Flunitrazepam |
| GABA -B | 8.19 | (GABA) | [^3^H]GABA |
| Glutamate (Non-selective) | 1.91 | (L-Glutamic acid) | [^3^H]Glutamic acid |
| Glutamate (AMPA) | 3.27 | (AMPA) | [^3^H]AMPA |
| Glutamate (Kainate) | 1.50 | (Kainic acid) | [^3^H]Kainic acid |
| Glutamate (NMDA agonist site) | 8.17 | (Glutamic acid) | [^3^H]CGP-39653 |
| Glutamate (NMDA glycine site) | 4.02 | (MDL105519) | [^3^H]MDL105519 |
| Glutamate (NMDA phencyclidine site) | 3.80 | ((+)-MK-801) | [^3^H]MK-801 |
| Glycine (Strychnine sensitive) | 0.00 | (Strychnine) | [^3^H]Strychnine |
| Histamine H1 (Central) | 1.83 | (Pyrilamine) | [^3^H]Pyrilamine |
| Histamine H2 | 3.80 | (Cimetidine) | [^3^H]Cimetidine |
| Histamine H3 | 0.00 | ((R)(-)-α-methyl histamine) | [^3^H]N-methyl histamine |
| Muscarinic M1 | 8.35 | (Atropine) | [^3^H]Pirenzepine |
| Muscarinic M2 | 16.31 | (Atropine) | [^3^H]AF-DX-384 |
| Nicotinic Ni | 2.10 | ((±)-Nicotine) | [^3^H]Nicotine |
| Opiate (Non-selective) | 43.44 | (Naloxone) | [^3^H]Naloxone |
| Serotonin 5-HT1A | 35.22 | (Serotonin) | [^3^H]8-OH DPAT |
| Serotonin 5-HT2A | 30.56 | (Ketanserin) | [^3^H]Ketanserin |
| Serotonin 5-HT3 (Human) | 7.36 | (MDL72222) | [^3^H]GR65630 |
| Sigma (Non-selective) | 24.20 | (Haloperidol) | [^3^H]DTG |

Test substance concentration: 1 μM, Positive substance concentration : 10 μM

Data are expressed as the mean values of duplicate samples.

The % inhibition was calculated from “100 – binding ratio”.

Binding ratio: [(B - N) / (B_0_ - N)] × 100 (%) B: Bound radioactivity in the presence of test substance and positive substance (individual value), B_0_: Total bound radioactivity in the absence of test substance and positive substance (mean value), N: Non-specific bound radioactivity (mean value)
