# Supplementary figures and images for: Pharmacological Characterization of [3H]CHIBA-3007 Binding to Glycine Transporter 1 in the Rat Brain
Source: PLoS One. 2011 Jun 23;6(6):e21322. doi: 10.1371/journal.pone.0021322 (PMC3121759; doi:10.1371/journal.pone.0021322)

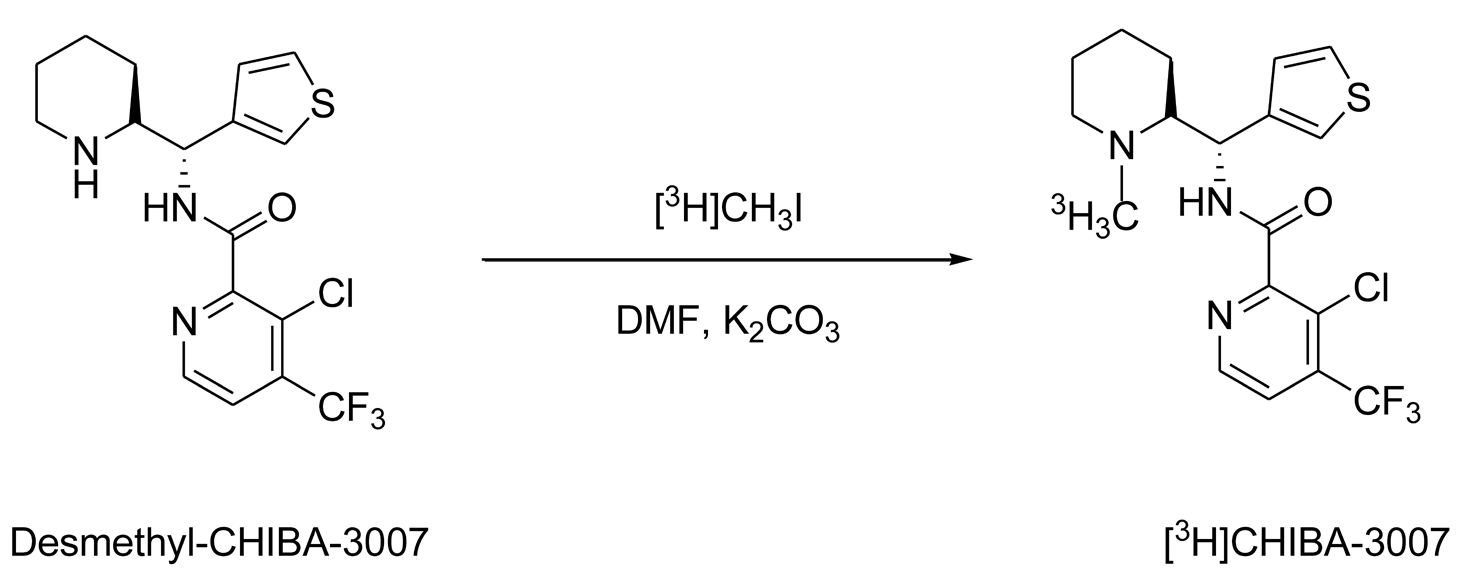

Supplement: Figure S1 — Preparation of [3H]CHIBA-3007. (TIFF) [file pone.0021322.s002.tiff]
